# Role of HCV Core gene of genotype 1a and 3a and host gene Cox-2 in HCV-induced pathogenesis

**DOI:** 10.1186/1743-422X-8-155

**Published:** 2011-04-01

**Authors:** Shah Jahan, Saba Khaliq, Bushra Ijaz, Waqar Ahmad, Sajida Hassan

**Affiliations:** 1 Applied and Functional Genomics Laboratory, National Centre of Excellence in Molecular Biology, University of Punjab, Lahore 53700, Pakistan

## Abstract

**Background:**

Hepatitis C virus (HCV) Core protein is thought to trigger activation of multiple signaling pathways and play a significant role in the alteration of cellular gene expression responsible for HCV pathogenesis leading to hepatocellular carcinoma (HCC). However, the exact molecular mechanism of HCV genome specific pathogenesis remains unclear. We examined the *in vitro *effects of HCV Core protein of HCV genotype 3a and 1a on the cellular genes involved in oxidative stress and angiogenesis. We also studied the ability of HCV Core and Cox-2 siRNA either alone or in combination to inhibit viral replication and cell proliferation in HCV serum infected Huh-7 cells.

**Results:**

Over expression of Core gene of HCV 3a genotype showed stronger effect in regulating RNA and protein levels of Cox-2, iNOS, VEGF, p-Akt as compared to HCV-1a Core in hepatocellular carcinoma cell line Huh-7 accompanied by enhanced PGE2 release and cell proliferation. We also observed higher expression levels of above genes in HCV 3a patient's blood and biopsy samples. Interestingly, the Core and Cox-2-specific siRNAs down regulated the Core 3a-enhanced expression of Cox-2, iNOS, VEGF, p-Akt. Furthermore, the combined siRNA treatment also showed a dramatic reduction in viral titer and expression of these genes in HCV serum-infected Huh-7 cells. Taken together, these results demonstrated a differential response by HCV 3a genotype in HCV-induced pathogenesis, which may be due to Core and host factor Cox-2 individually or in combination.

**Conclusions:**

Collectively, these studies not only suggest a genotype-specific interaction between key players of HCV pathogenesis but also may represent combined viral and host gene silencing as a potential therapeutic strategy.

## Background

An estimated 3% of the world's population is chronically infected with Hepatitis C virus (HCV) which is the main cause of liver fibrosis and cirrhosis, often leading to HCC (hepatocellular carcinoma) in a substantial number of patients. Almost 10 million people in Pakistan are living with HCV [1,2]. The most prevalent HCV genotype in Pakistan is 3a followed by 1a [3]. HCV virion is enveloped and has a positive strand genome comprising 9.6 kb RNA which is processed by cellular and viral proteases into 10 viral proteins, Core, E1, E2, p7 (structural proteins), NS2, NS3, NS4a, NS4b, NS5a and NS5b (nonstructural proteins). Although specific mechanisms by which HCV disease progresses remains unknown, direct interaction of specific viral proteins with host cell system has shown to be accounted for some of its pathophysiological profile of HCV patients [4].

Besides nucleocaspid formation, HCV Core protein, in particular, also modulates gene transcription, cell proliferation, cell death and interferes with metabolism leading to oxidative stress, liver steatosis and eventually HCC. Oxidative stress has emerged as a key contributor in the development and progression of HCV-induced pathogenesis of liver [5,6]. Several factors might contribute to oxidative stress associated with HCC, as HCV Core and NS5a proteins, which are able to up-regulate Cox-2 expression; a key player of oxidative stress in hepatocytes derived cells [7]. The expression of Cox-2 in HCC was found to correlate with the levels of several key molecules implicated in carcinogenesis such as iNOS (induced nitric oxide synthase), VEGF (vascular endothelial growth factor) and p-Akt [8,9]. iNOS and Cox-2 have carcinogenic effects achieved either directly or by producing mediator that regulate cellular growth [10]. Cox-2 can induce angiogenesis growth factors via VEGF in HCV associated HCC [9,11,12]. A number of other inflammatory mediators including nitric oxide (NO), Cox-2, and prostaglandin E2 (PGE2) in cultured hepatocellular carcinoma cells also stimulate VEGF [11]. Cox-2 activates Akt in human HCC via a p13-kinase-dependent mechanism [13], it acts as an important signal mediator, which regulates cell survival and proliferation [14,15]. Despite several studies, mechanisms involved in HCV-associated pathogenesis are not completely understood; it may vary as a function of viral genotype [16].

Very few studies have been undertaken to evaluate the role of HCV Core protein of genotype 3a and 1a in HCV induced pathogenesis. In this study, we selected Huh-7 cells as culture model system for the transient transfection using HCV 3a and 1a Core genes and viral load analysis using HCV-infected serum as inoculum. We found a greater effect of HCV Core of genotype 3a on the expression levels of Cox-2, iNOS, VEGF, p-Akt in transiently expressing Core protein and in serum-infected Huh-7 cells as well as in patient's blood and liver biopsies samples. Although, HCV Core protein is highly conserved between different genotypes, we observed differences in the HCV Core region of 1a and 3a and speculate that these differences may differentially regulate hepatic pathogenesis in HCV infected individuals.

HCV RNA is highly susceptible to RNAi-induced suppression, as many investigators have reported the inhibition of HCV RNA levels by targeting different genes using RNAi. In our previous study, we used siRNA against HCV 3a Core and inhibited its expression not only in transiently transfected cells but also in serum infected Huh-7 cells [17]. Here, we used siRNA against Core and Cox-2 genes either alone or in combination with HCV 3a Core gene-transfected and serum-infected Huh-7 cells to further study HCV-induced pathogenesis. We observed additional inhibitory effect of HCV Core and Cox-2 silencing on the expression levels of genes involved in HCV pathogenesis, suggesting a possible virus host interaction among these, moreover, reduction of viral titer may also represent potential combinational therapeutic approach against HCV infection.

## Results

### Role of HCV Core in HCV induced pathogenesis

**Figure 1:**
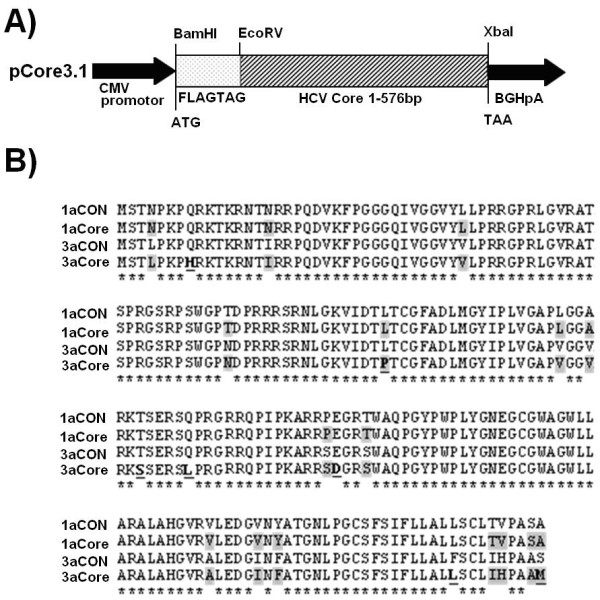
**HCV Core expression plasmids and protein sequences comparison of HCV genotype 1a and 3a**. A) Schematic diagram of HCV Core expression plasmid illustrating the FLAG tag sequences having ATG start site and with HCV3a and 1a Core (1-573 bp) sequence insertion into mammalian expression plasmid pCR3.1. The CMV promoter and BGHpA regions of pCR3.1 are also shown. (B) Amino acid sequence comparison of HCV Core 1a and 3a clones. Comparison of full predicted amino acid sequence of HCV Core isolates in our study against a consensus genotype 3a clone (3aCON) and 1a (1aCON) from the HCV sequence database, performed using the ClustalW program. Sequences different from consensus are boldface and underlined. Differences in the individual amino acid Core 1a and Core 3a are highlighted. Asterisks at the bottom of a sequence comparison indicate complete identity at that position.

**Figure 2:**
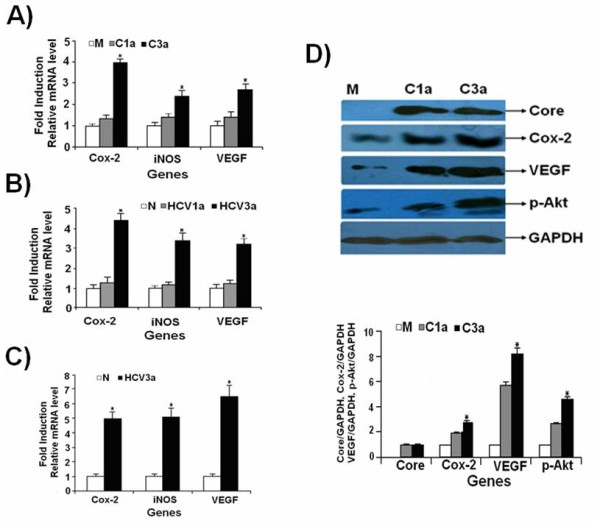
**HCV 3a Core gene shows higher induction of Cox-2, iNOS, VEGF and p-Akt compared to HCV 1a Core gene**. (A) Comparison of gene expression of Cox-2, iNOS and VEGF in Huh-7 cells transiently transfected with HCV Core 1a (C1a), 3a (C3a) and pCR3.1 plasmid alone as mock (M) samples (0.4 μg DNA/well of each plasmid). After 48 hrs incubation, relative gene expression levels were determined. (B) Comparison of expression of Cox-2, iNOS, and VEGF genes expressed as relative fold induction in HCV 1a and 3a infected patient's blood samples verses normal. (C) Expression of Cox-2, iNOS and VEGF genes in HCV 3a genotype infected liver biopsies verses normal. (D) The protein expression levels were determined by Western blot analysis of Huh-7 cell lysates transfected with HCV Core vectors 1a, 3a and effect on Cox-2, VEGF, p-Akt and GAPDH using specific antibodies. Blots were also normalized by measuring the amount of GAPDH (histogram). Bars are mean of optical density ratio of each group.* p < 0.001 verses control and Core 1a. GAPDH gene was used as internal control for normalization in Real-Time PCR and in Western blotting. All experiments were performed in 3 independent experiments having triplicate samples in each.

HCV 1a and 3a Core genes were cloned into pCR3.1/FLAG tag (Figure 1A). The amino acid sequence comparison showed that 3a Core protein has 90% similarity to 1a Core protein and 98% similarity to their consensus sequences (Figure 1B). We observed several nucleotide differences in both genotypes when compared with literature. These changes are N in 1a at position 4 and L in 3a, Q in 1 and H in 3 at position 8, N in 1 I in 3a at position 16 and T in 1a and N in 3a at position 70. The other reported difference is YATG in 1a and FATG in 3a at position 164 amino acid (Figure 1B). In order to observe whether 1a and 3a differentially regulate the expression of Cox-2, iNOS, p-Akt and VEGF, Huh-7 cells were transiently transfected with or without 0.4 μg HCV genotype 1a and 3a Core expressing plasmids for 48 hrs. The mRNA expression levels of Cox-2, iNOS, and VEGF genes were determined using Real-Time PCR. As shown in Figure 1C, HCV 3a Core induced the expression of Cox-2, iNOS and VEGF genes at significantly higher levels as compared to HCV 1a Core gene. Up-regulated levels for Cox-2, iNOS and VEGF were 4.1, 2.4 and 2.8-fold, respectively, with HCV 3a Core whereas; HCV 1a Core induced Cox-2 (1.3-fold), iNOS (1.2-fold) and VEGF (1.3-fold) as compared to control (Figure 2A). Similar results were obtained with HCV 1a and 3a infected patient's PBMCs. Up-regulation of genes in HCV 3a infected blood was as follow: Cox-2 (4.19-fold), iNOS (3.4-fold), and VEGF (3.22-fold) as compared to HCV 1a infected patient's blood and normal samples (Figure 2B). In HCV 3a infected biopsy samples, the expression levels for these genes were in this fashion: Cox-2 (4.97-fold), iNOS (5.08-fold), and VEGF (6.49-fold) compared with normal samples (Figure 2C). Cell lysates from Core transfected Huh-7 cells were examined by Western blot analysis using Core, Cox-2, VEGF, and p-Akt specific antibodies. Significant increase (p < 0.001) in the protein expression levels in HCV 3a genotype was observed as compared to HCV 1a (Figure 2D).

**Figure 3:**
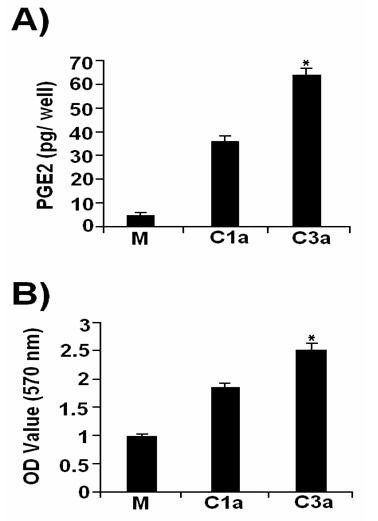
**Enhanced expression of PGE2 and cell proliferation in Core transfected Huh-7 cells**. (A) Prostaglandin E2 production in response to HCV 1a and 3a Core transfected Huh-7 cells were measured with Biotrak Prostaglandin E2 Enzyme Immunoassay system. (B) MTT assay shows that HCV 3a Core significantly increased the proliferation of Huh-7 cells as compared to mock and Core 1a transfected cells, observed at 570 nm wavelength. All experiments were performed in 3 independent experiments having triplicate samples in each. Error bars indicate, mean S.D, *p < 0.01 verses mock.

In order to determine the effect of HCV Core gene expression on cell proliferation, PGE2 levels were determined by Biotrak Prostaglandin E2 Enzyme Immunoassay system. HCV Core protein of both genotypes significantly increased the production of PGE2 more with HCV 3a Core than HCV 1a in Huh-7 cells (Figure 3A). Core induced cell proliferation was also determined through MTT cell proliferation assay. These results mirrored those observed with PGE2, a significant enhanced cell proliferation observed with both genotypes. HCV 3a genotype has greater effect compared with HCV 1a genotype (Figure 3B). Taken together, these results show a genome-specific involvement of HCV Core in the induction of Cox-2, iNOS, p-Akt and VEGF host genes at RNA/protein levels in Huh-7 cells. Their high expression in HCV 3a infected patient's samples and cell proliferation assay/PGE2 levels further supported genome-specific role of HCV in its pathogenesis.

### Effect of HCV 3a Core-specific siRNA on the expression of Cox-2, iNOS and VEGF genes

**Figure 4:**
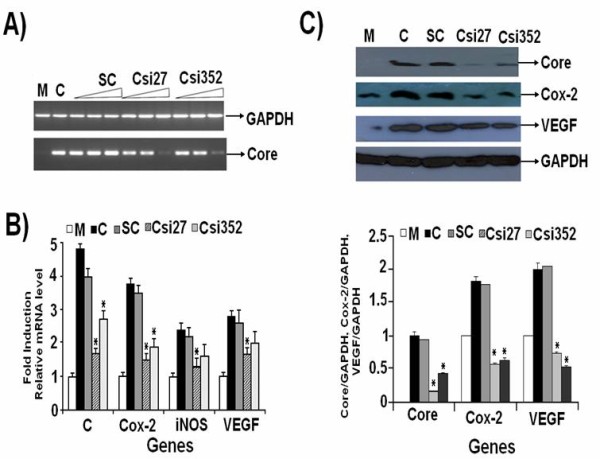
**HCV 3a Core-specific siRNA inhibit expression of genes involved in HCV pathogenesis**. Huh-7 cells were transfected with HCV 3a Core expression vector or mock along with or without siRNA. (A) Dose dependent mRNA expression of HCV 3a Core gene as a result of 10 μM, 20 μM and 40 μM of siRNAs for 48 hrs. Cells were harvested and relative RNA levels in PCR Csi27 and Csi352 siRNAs tranfected cells were determined using semi-quantitative PCR. Expression levels for mock-transfected (M), HCV 3a Core expression plasmid (C), scramble siRNA (SC) and GAPDH are also shown. (B) Silencing effect of HCV 3a Core gene on the RNA expression levels of cellular genes (Cox-2, iNOS and VEGF) 48 hrs post transfection on Real-Time PCR using gene specific primers in comparison to mock was performed. GAPDH was used as internal control. Three independent experiments were performed having triplicate samples. Error bars indicate, mean S.D, *p < 0.001 verses Core. (C) Silencing of HCV 3a Core gene and its effect on Cox-2 and VEGF protein level using specific antibodies were determined by Western blot analysis after 48 hrs transfection in Huh-7 cells with GAPDH as internal control. Bars are mean of optical density ratio of each group.* p < 0.001 verses control and Core 3a.

**Figure 5:**
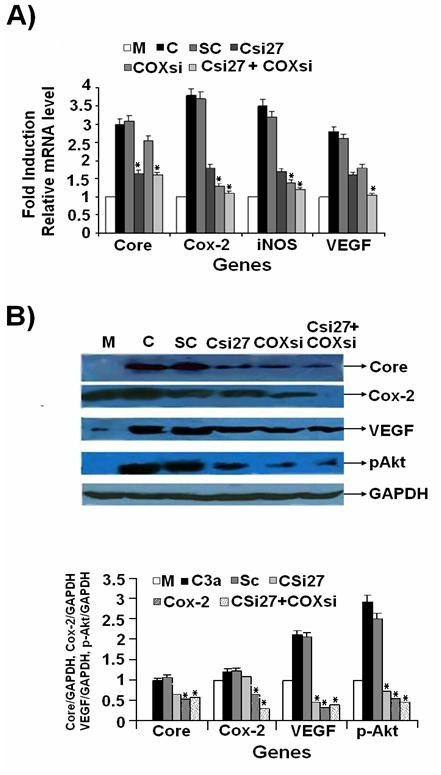
**Combined effect of Cox-2 and HCV 3a Core siRNAs on Cox-2, iNOS, VEGF and p-Akt gene expression**. (A) Huh-7 cells were transfected with HCV 3a Core expression vector (C) or mock-treated (M) along with or without siRNAs (Csi27 and COXsi) alone or in combination (Csi27+COXsi) for 48 hrs. Total RNA was quantified by Real-Time PCR and is shown as fold induction for Cox-2, iNOS and VEGF genes using their gene specific primers. (B) Western blot analysis of Huh-7 cells treated with and without Core, Cox-2 and combined siRNA was carried out using specific antibodies. Three independent experiments with triplicate determinations were performed. Error bars indicate mean S.D, * p < 0.01 verses serum 1a.

HCV 3a Core showed significantly higher induction levels as compared to HCV 1a Core. Therefore, we utilized gene silencing against HCV 3a Core to investigate its involvement in the regulation of Cox-2, iNOS, p-Akt and VEGF genes. Transient transfection of two in-vitro transcribed siRNAs Csi27 and Csi352 in Huh-7 cells after 48 hrs showed a dramatic reduction of mRNA expression levels of HCV 3a Core gene in a dose-dependent manner. Maximum inhibition of 3-fold and 2-fold was observed for Csi27 and Csi352 at 40 μM concentrations, respectively, when compared with scramble siRNA (Figure 4A). The effect of HCV 3a Core siRNAs on mRNA expression levels of Cox-2, iNOS, VEGF genes in Huh-7 cells was also determined. Although, both Csi27 and Csi352 siRNAs decreased the expression levels of all four genes, Csi27 showed dramatic reduction for Cox-2 (2.5-fold) gene, whereas, expression of iNOS, and VEGF was reduced to 1 fold, respectively (Figure 4B). Complementary to reduced mRNA levels, HCV 3a Core protein expression was also inhibited 90% and 76% with Csi27 and Csi352 siRNA, respectively. These siRNA also inhibited protein expression levels of Cox-2 (85% with Csi27 and 70% with Csi352) and VEGF (75% with Csi27 and 65% with Csi352) as compared to control scramble siRNA (Figure 4C). Similarly, the expression of Cox-2 gene was reduced to the basal levels with its own siRNA, whereas, Cox-2 siRNA treatment decreased the expression levels of iNOS and VEGF genes to 2 and 3-fold, respectively (Figure 5A).

### Inhibition of genes with combined silencing of HCV 3a Core and host Cox-2 genes

**Figure 6:**
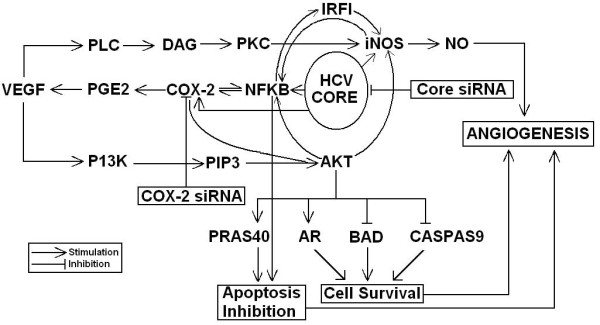
**HCV Core protein affects on the cellular genes involved in oxidative stress, apoptosis, cell survival and angiogenesis**. Schematic diagram illustrating the role of HCV Core on cellular genes. In HCV, normal angiogenesis process can be malignant by deregulation of genes involved in this pathway. HCV Core protein up-regulates NFKB, which further up-regulates iNOS and COX-2 pathways, and inhibit apoptosis by blocking bad and caspase pathway. COX-2 up-regulation leads to apoptosis inhibition via VEGF pathway. VEGF can trigger angiogenesis either by iNOS or AKT activation. AKT singling also up-regulates AR and inhibit BAD and Caspase9 that leads to angiogenesis via cell survival. AKT also have direct impact on iNOS regulation that also escorts to angiogenesis. We corroborated that using siRNA against Core, or Cox-2 either alone or in combination can block angiogenesis directly or via Cox-2 reduced expression. PLC, *PhosphoLipase*; DAG, diacylglycerol; PKC, protein kinase C; iNOS, inducible nitric oxide synthase; NO, nitric oxide; VEGF, vascular endothelial growth factor; PGE2, prostaglandin E2; COX-2, cyclooxygenase-2; NFKB, nuclear factor kappa-B; P13K, phosphoinositide-3-kinase; PIP3, phosphatidylinositol 3,4,5-trisphosphate; PRAS40, proline-rich Akt substrate of 40 kDa; AR, Aldose reductase; BAD, Bcl-XL/Bcl-2-associated death promoter; CASPASE9, apoptosis-related cysteine peptidase.

We further investigated whether HCV Core 3a is regulating gene expression directly or via Cox-2. HCV Core 3a transfected cells were treated with Core and Cox-2 specific or scrambled siRNAs either alone or in combination. Real-Time PCR results showed that Core-induced expression of Cox-2, iNOS and VEGF genes was markedly reduced to 3, 2 and 1.5 fold respectively, after co-treatment with HCV 3a Core and Cox-2 specific siRNA (Figure 5A). A dramatic reduction was observed at protein levels for Cox-2, VEGF and p-Akt in cells treated with Core and Cox-2 siRNA in combination (Figure 5B). The over all mechanism of regulation of oxidative stress by HCV Core and Cox-2 has been shown in Figure 6.

### Suppression of viral load and gene expression by HCV Core and Cox-2 siRNA in Huh-7 cells

**Figure 7:**
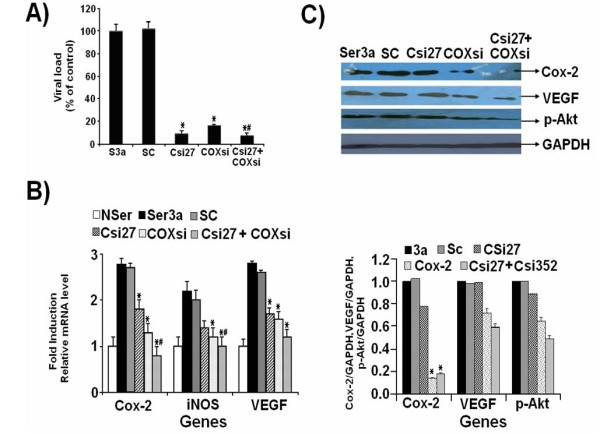
**Silencing of Cox-2 and HCV 3a Core genes either alone or in combination and reduction of viral titer in Huh-7 cells infected with HCV 3a sera**. (A) After 3 days of post infection of Huh-7 cells with HCV 3a sera (Ser 3a), cells were transfected with siRNAs (Csi27 and COXsi) either alone or in combinations (Csi27+COXsi). Data are expressed as mean percent viral load of non-siRNA treated samples. (B) Gene expression analysis was performed using Real-Time PCR. The effect of siRNA on the induction of genes in serum infected and siRNA treated Huh-7 cells. Data are expressed as relative fold reduction to mock sample for Cox-2, iNOS and VEGF genes. (C) The effect of siRNA on protein expression levels of Cox-2, VEGF, p-Akt and GAPDH were determined after 72 hrs post infection with HCV 3a Serum by Western blot analysis from Huh-7 cell lysates using specific antibodies. Three independent experiments with triplicate determinations were performed. Error bars indicate, mean S.D. *#p < 0.001 verses Csi27. * p < 0.001 verses Ser3a.

Additionally, the efficacy of siRNA against HCV 3a Core and Cox-2 genes alone or in combination was evaluated in serum-infected Huh-7 cells. A remarkable suppression of HCV RNA was observed up to 92% with HCV 3a Core-siRNA, 83% with Cox-2 siRNA and 95% with combination of both siRNAs (Figure 7A). Both siRNA either alone or in combination inhibited the RNA expression levels of Cox-2, iNOS and VEGF. These results indicate that siRNA against HCV 3a Core and Cox-2 genes decreased the mRNA expression levels of Cox-2 (2-fold), iNOS (1.5-fold) and VEGF (1.8-fold) genes (Figure 7B). Additionally, there was significant reduction at protein levels of Cox-2, VEGF and p-Akt in cells treated with Core and Cox-2 siRNA (Figure 7C).

## Discussion

HCV-induced pathogenesis like liver injury, insulin resistance and steatosis may be genotype specific. A high rate of HCV associated HCC prevalence in Asia has been observed with genotype 3 followed by 1 responsible for most of the cases in Pakistan [3,18-20]. Several studies have been conducted to unravel the HCV pathogenesis related to genotype 1 and 2 [20,21]. However, no studies related to role of HCV genotype 3a in oxidative stress are currently available. In this study, we compared the expression of HCV genotype 1a and 3a in oxidative stress.

Our results showed a significantly higher expression of Cox-2, iNOS, and VEGF genes in HCV 3a patient blood as compared with HCV 1a genotype at mRNA levels. We observed the significant stimulation of Cox-2, iNOS, and VEGF in HCV 3a infected patients biopsy samples compared to normal. Unfortunately, we were unable to compare the expression level of these genes in HCV infected 1a patients biopsy samples because of non-availability of 1a liver biopsy samples as very few patients agreed to underwent liver biopsy. The expression levels of Cox-2, iNOS, p-Akt and VEGF are positively correlated in cultured cells and in human liver cancer tissues suggesting their involvement in chronic liver diseases. NO and Cox-2 have carcinogenic effects achieved either directly or by producing mediators that regulate cellular growth [9,10]. Over expression of Cox-2 induces angiogenesis through increased levels of proinflammatory molecule PGE2 which in return activates VEGF [22] and Akt that promote the growth of human HCC cells [9,13].

HCV Core protein is involved in a whole array of host cell functions including signal transduction and transcriptional regulation of genes in the liver (5). It is reported that HCV Core protein regulates Cox-2 expression in hepatocytes and causes oxidative stress leading to HCC [7]. In the present study, Core protein of HCV 3a was found to enhance the expression of cellular genes Cox-2, iNOS, p-Akt and VEGF at mRNA and protein at higher level compared to Core gene of HCV 1a in Huh-7 cell line. The production of PGE2 was assayed in HCV 1a and 3a transfected cells and results indicated that the level of PGE2 was increased in HCV 3a Core transfected cells as compared to 1a. Furthermore, cell proliferation was found to be enhanced in Core 3a transfected cells because of Core-induced Cox-2 activation of PGE2 release at 48hrs post transfection (Figure 3). Our results support the observation that enhanced synthesis of Cox-2 as a consequence of Core 3a induced PGE2 that stimulates the release of proangiogenic factor VEGF. VEGF production is regulated by COX-2, suggesting a promoting role of COX-2 in tumor angiogenesis through the COX-2/VEGF system [[20,23] and [24]].

Many studies have reported that substitutions in the HCV Core region results in enhanced insulin resistance, steatosis, oxidative stress and hepatocellular carcinoma [25,26]. Amino acid substitution at the sequence YATG (1b) and FATG (3a) of HCV Core gene was found to be important for FAS activation in genome specific manner [21]. Previously, Hourioux *et al*., (2007) showed a greater involvement of these HCV 3a amino acid sequences in lipid accumulation and steatosis in cell culture system [27]. Furthermore, Jhaveri *et al*., (2008) reported that amino acid substitution of HCV 3a Core (L**L**SCL**I**H) at 182 and 186 positions are associated with HCV induced steatosis [28]. Amino acid substitution in HCV Core gene has different impact in hepatic steatosis and oxidative stress in chornic HCV patients. Tachi *et al *(2009) has reported that in HCV genotype 1b infected patients with hepatic steatosis, there was amino acid substitution 'Q' at 70 while in patients without steatosis Arg was present. He also reported that Met91aa of Core region in patients infected with HCV 1b have high oxidative stress measured through the level of 8-hydroxy-20-deoxyguanosine as compared in those with Leu91aa [20]. Mann *et al*., observed that the divergence in Core 9-11 amino acid at genotype level modulate the NF-kB activation, a transcription factor which play important factor in inflammation and liver injury. Core encoding RKP failed to activate NF-kB signaling in vitro while NF-kB activation by Core encoding RQT does not differ from control RKT Core [29]. Steatosis and oxidative stress are interlinked and arise due to accumulation of triglyceride rich lipids in cytoplasm [4,30]. We have also observed the same amino acid variations that were previously reported plus few other substitutions in HCV 3a Core sequence in comparison with 1a (Figure 1B). Although there is 90% amino acid sequence similarity, however, we observed few sequence differences too. These changes are N in 1a at position 4 and L in 3a, Q in 1 and H in 3 at position 8, N in 1 I in 3a at position 16 and T in 1a and N in 3a at position 70. Therefore, we speculate that these sequences may play a role in differentially regulating genes involved in oxidative stress leading to HCC. Vidali *et al*., found a relationship between oxidative and hepatic steatosis in the progression of chronic hepatitis C [16]. Several recent studies have shown variable responses for interferon IFN-ribavirin combination therapy, oxidative stress/steatosis and insulin resistance due to the amino acid substitutions in the HCV Core region [26,31-33]. Since, there is no previous report linking amino acid variations to oxidative stress in a genome-specific manner, additional studies are needed to identify the contribution of these substitutions to the HCV induced pathogenesis.

We silenced Core gene using siRNA to further confirm the role of HCV 3a Core gene in HCV infection and stimulation of cellular genes. RNAi activity directed against multiple target sequences of the HCV genome has been found to block effectively the synthesis of replicon RNA [17,34]. We observed efficient inhibition of HCV Core by siRNA against this functionally important region of HCV 3a genotype. Protein expression analysis revealed suppression of HCV Core protein levels by 90% in Csi27, while suppression by Csi352 siRNA was 76% (Figure 4). Two different groups have used siRNA against Core gene of HCV 1a and 1b genotype and observed 60% and 80% reduction in mRNA and protein expression, respectively [34,35]. Silencing of HCV Core through Core specific siRNA resulted in reduced expression of host cellular genes Cox-2, iNOS and VEGF in Core transfected Huh-7 cells at both mRNA and protein levels. The significant inhibition of Core mRNA in siRNA transfected cells also resulted in 50% reduction in PGE2 production and cell proliferation. The ability of Core siRNAs to inhibit HCV expression correlates well with the established role of HCV 3a Core gene in oxidative stress and angiogenesis by regulating these genes.

HCV Core up-regulate Cox-2 levels that correlated with genes involved in HCV pathogenesis. Since the inhibitors of Cox-2, such as siRNAs and celecoxib showed an inhibition of cell proliferation and carcinogenesis, we used Cox-2 specific siRNA to determine its effect on gene expression. Cox-2 specific siRNA (COXsi) significantly decreased the expression of not only the Cox-2 itself but also the expression levels of p-Akt, iNOS and VEGF. These results suggest that Cox-2 pathway is involved in mediating the activation of p-Akt, iNOS and VEGF in HCV Core expressing cells (Additional file 1 and Additional file 2, Figure S1).

Additionally, we explored whether HCV Core induced Cox-2 gene, which in turn activated Akt (phosphrylation), iNOS, and VEGF or it has a direct role in activating cellular genes understudy. We observed significant reduction in the expression of Core and these genes when treated with Csi27 and COXsi in combination at both mRNA and protein levels (Figure 5). The significant inhibition of Core and Cox-2 mRNA in siRNA transfected cells either alone or in combination also resulted in reduced PGE2 production and cell proliferation to 50%, 65% and 85%, respectively (Additional file 1 and Additional file 3, Figure S2). The reduction of these genes by both HCV Core and Cox-2 siRNA indicate that HCV Core is involved in HCV pathogenesis not only by exerting its effect through Cox-2 signaling but is also directly involved in the regulation of gene expression. Zhao *et al*., (2007) observed reduced production of PGE2 and VEGF level in treated Huh-7 cells, and suppressed HCC-associated angiogenesis *in vitro *and *in vivo *using Cox-2 siRNA [20].

Most of the studies are conducted in Huh-7 derived cell lines and with replicons supporting HCV RNA transcription and protein synthesis. Recently, different groups have studied the HCV replication in serum infected liver cell lines [17,36-39]. In order to confirm above results, we infected Huh-7 cells with native viral particles from HCV genotype 3a positive serum, the most prevalent type in Pakistan using already established protocol [37,40]. Previously in our laboratory, Khaliq *et al*., 2010 infected Huh-7, HEK, MDBK and HeLa cell line with HCV 3a infected serum and observed that MDBK and HeLa cell lines did not support HCV infection [41].

The current therapies against HCV have limited efficacy due to the development of viral resistance and HCV high mutation rate. The problem of viral mutants could be resolved by using a mixture of siRNAs against different sequences. Several studies have also demonstrated the feasibility of targeting host cellular and viral factors involved in HCV infection as potential targets for therapy either alone or in combinations of both like Lamin A/C, Caspase-8, Hsp90 and HCV genes [42-47]. The effect of HCV sera on the expression of Cox-2, iNOS and VEGF genes and their inhibition by siRNA against HCV Core and host gene Cox-2 was also observed. The expression of Cox-2, iNOS and VEGF genes both at mRNA and protein levels were found to be more stimulated in Huh-7 cells infected with HCV 3a serum as compared to HCV 1a (Data shown in Additional file 1 and Additional file 4, Figure S3). These results were consistent with our HCV Core over-expression studies. We observed HCV replication in the Huh-7 cells through detection of 5'UTR by Real-Time PCR in cells at 3^rd ^and 5^th ^day post-infection and 92% reduction with Csi27, 83% with COXsi and 95% inhibition when both siRNAs were used (Figure 7). At the day 3^rd ^post infection, we observed that the decline of viral titer using Core and Cox-2 siRNA in liver cells also reduced the expression of Cox-2, pAkt, iNOS and VEGF. Zekri *et al*., has reported that siRNA against HCV Core and 5'UTR effectively blocked HCV replication in serum infected Huh-7 cells [40]. Many authors have observed and also correlated amino acid substitution in Core in HCV infected patient's samples with steatosis, interferon response, insulin resistance and oxidative stress. HCV structural proteins play a direct role in the development of liver steatosis and increase the risk of liver cancer in transgenic mice [48-50]. In transgenic mouse model, expressing HCV structural proteins produced profound liver damage leading to hepatocellular carcinoma. Moriya et al., (1997) reported that transgenic mouse model expressing HCV Core protein showed hepatic tumor, lipid accumulation in 16 months old mice [51]. Transgenic mice with steatosis displayed dysplastic growth of cells evolving into tumor formation. Furthermore, transgenic mice showed deposition of collagen and progressive fibrosis [52]. In our study, we observed high expression of oxidative stress related genes in patients infected with HCV genotype 3a as compared to 1a. In future, the correlation of amino acid substitutions are need to be address in transgenic mouse model or the other natural victim of HCV that is chimpanzee to confirm our results and the full understanding of the pathogenesis.

## Conclusions

In conclusion, these studies for the first time suggest an interaction of HCV 3a Core and Cox-2 in regulating the genes involved in HCV pathogenesis which may provide a better understanding for genome-specific mechanisms involved in disease progression regulating Cox-2, pAkt, iNOS, PGE2 and VEGF expression directly or indirectly. Another outcome of these studies is the viral titer reduction observed with combined gene silencing of HCV Core and Cox-2, which support the idea of dual strategy against both the viral and host genes to be a potent approach in the treatment of chronic hepatitis C.

## Methods

### Patient's Samples

The local HCV 3a and 1a patient's serum samples used in this investigation were collected from CAMB (Center for Applied Molecular Biology) Diagnostic Laboratory, Lahore, Pakistan after clinical diagnosis. ELISA and PCR positive samples were stored at -80°C prior to RNA extraction. The genotyping and quantification was performed as previously described by Ahmad *et al*., (2010) [3]. None of the subjects had received antiviral therapy. Subjects with clinical evidence of HBV, diabetes and any other type of cancer were excluded from study. The subject population with chronic HCV infection, viral load > 2×10^8 ^IU/ml of genotype 1a and 3a were included in this study. Blood samples from three control and six HCV positive patients for viral serum inoculations were taken and immediately stored at -20°C. For expression of oxidative stress related genes, three normal and six HCV positive liver biopsies and their blood samples were also collected from Jinnah Hospital Lahore, Pakistan. Study was approved from the Institutional Ethical Committee and patient's written consent was obtained.

### RNA Isolation from Blood and Biopsy Samples

Total RNA from liver biopsies was extracted by using Qiagen RNeasy Mini kit (Qiagen, USA). For PBMCs, TRIzol reagent (Invitrogen, Carlsbad, CA) was used according to manufacturer's protocol. Briefly, first the white blood cells (PBMC) were separated from blood collected in anticoagulant (EDTA) tube. For the lysis of erythrocytes 900 μl of RBC lysis solution (Gentra USA) was added in 300 μl of whole blood. The resulting mixture was incubated at room temperature for 10 minutes and then, centrifuged at 1000 rpm for 1 minute at 4°C. The same step was repeated unless PBMC formed a tight white pellet at the bottom of the tube. Then Trizol reagent (Invitrogen, Carlsbad, CA) was used for the isolation of RNA from these cells according to manufacturer's protocol.

### Plasmid Construction

Viral RNA was isolated from thawed 100 μl serum aliquots using Gentra RNA isolation kit (Gentra System Pennsylvania, USA) according to the manufacturer's instructions. 100-200 ng extracted viral RNA was used for RT PCR using the SuperScript III one step RT-PCR system (Invitrogen). HCV complementary DNA (cDNA) encoding the full length Core protein (amino acid 1-191 of HCV isolates of HCV 3a genotype) were amplified using Core specific primers. The PCR conditions were 94°C 3 min; 94°C 35 sec; 58°C 45 sec; 72°C 1 min for 35 cycles and final extension at 72°C for 10 min. PCR product was cloned into pCR3.1 mammalian expression plasmid with FLAG tag inserted at the 5' end with restriction sites *EcoRV *and *Xba1 *(Fermentas, USA). Similarly, The plasmid HFL (a kind gift provided by Dr. Zafar Nawaz University of Miami, USA) containing full length of HCV 1a was used as the template for PCR and later cloning of HCV 1a Core in pCR3.1 mammalian expression vector (Figure 1A). Sequencing was performed for both expression plasmids by DNA Core facility at CAMB, Lahore, Pakistan. Accession number for HCV 3a Core sequences submitted to NCBI is EU266536.

### Cell Culture

Huh-7 cell line (kindly provided by Dr. Zafar Nawaz, University of Miami, USA) was maintained in 75 cm^2 ^culture flasks (Iwaki, Japan) containing DMEM (Sigma Aldrich, USA) supplemented with 100 μg/ml penicillin/streptomycin, and 10% FBS as complete culture media (CCM) (Sigma Aldrich, USA), at 37°C with 5% CO_2 . _The CCM was renewed every 3^rd ^day and cells were passaged every 4-5 days. Viable cells were counted using 0.5% trypan blue (Sigma Aldrich, USA).

### Transient Transfection

Approximately 5×10^5 ^Huh-7 cells were seeded into 6-well tissue culture plates, at 24 hrs prior to transfection. Huh-7 cells were transiently transfected with and without expression plasmids (0.4 μg) of HCV Core 1a and 3a genotype in serum-free media using Lipofectamine™ 2000 (Invitrogen) according to the manufacturer's protocol. After 6 hours incubation at 37°C in 5% CO_2_, cells were washed with 1× PBS and CCM was added to the cells. Cells were harvested at 48 hrs post-transfection for genes expression analysis by Real-Time PCR and western blot analysis.

### Design and Synthesis of siRNA

The DNA sequencing facility at CAMB Lahore, Pakistan carried out sequencing of the Core gene of local HCV 3a patient's from serum samples. These sequences were aligned using the software CLUSTAL_W option of MEGA v.3.1. siRNA against HCV 3a Core and Cox-2 were designed to the most conserved target region using the Ambion's siRNA design tool http://www.ambion.com/techlib/misc/siRNA_tools.html (Table 1) and did not show any homology to other known human genes. A negative control siRNA (scrambled siRNA) was designed using scrambled sequences (Table 1). siRNA were synthesized using Ambion's siRNA silencer kit according to the manufacturer's instructions (Ambion, USA).

**Table 1:** Sequences of primers and target siRNAs

Primers used in vector construction	
Core3a-sense	ATGAGCACACTTCCTAAACC
Core3a-antisense	CATGGCTGCTGGATGAATTA
3aCore-sense	GCGATATCATGAGCACACTTCCTAAA
3aCore-antisense	AATCTAGATCATGGCTGCTGGATGAAT
1aCore-sense	GCGATATCAGCACGAATCCTAAAGGT
1aCore-antisense	AATCTAGATTAGG CTGAAGCGGGCACGGT

**Primers used in Real-Time PCR**	

RTp3.1-sense	GGACGACGATGACAAGGACT
RT1a-antisense	GGGGAGACAGGA GCCATC
RT3a-antisense	GGCTGTGACCGTTCAGAAGT
Cox-2-sense	AACCCACTCCAAACACAG
Cox-2-antisense	CTGGCCCTCGCTTATGATCT
iNOS-sense	CACCTTGGAGTTCACCCAGT
iNOS-antisense	ACCA CTCGTACTTGGGATGC
VEGF-sense	CTTGCCTTGCTGCTCTACC
VEGF-antisense	CACACAGGATGGCTTGAAG

GAPDH-sense	ACCACAGTCCATGCCATCAC
GAPDH-antisense	TCCACCACCCTGTTGCTGTA

**siRNAs used for silencing**	

Csi27-sense	AAGGATGGTGTTTCTTTTGGTCCTGTCTC
Csi27-antisense	AAACCAAAAGAAACACCATCCCCTGTCTC
Csi352-sense	AAATCGATGACTTTACCCAAACCTGTCTC
Csi352-antisense	AATTTGGGTAAAGTCATCGATCCTGTCTC
Scramble-sense	AAGTCGAGTCGCGTATGCAGGCCTGTCTC
Scramble-antisense	AACCTGCATACGCGACTCGACCCTGTCTC
COXsi-sense	AATTATTTCTGAAACCCACTCCCTGTCTC
COXsi-antisense	AAGAGTGGGTTTCAGAAATAACCTGTCTC

### Viral Inoculation

Huh-7 cell line was used to establish the *in vitro *replication of HCV. Cell culturing and viral inoculations were conducted as published previously [37,40]. High viral titer (> 1×10^8 ^IU/ml) from HCV 3a and 1a patient's was used as principle inoculums in these experiments. Huh-7 cells were maintained in 6-well culture plates, as described above, to semi-confluence in CCM, washed twice with serum-free medium, then inoculated with 500 μl (5 × 10^7 ^IU/well) of HCV 3a and 1a infected patient sera and 400 μl serum free media. Cells were maintained overnight at 37°C in 5% CO_2_. The next day, adherent cells were washed three times with 1× PBS, the CCM was added and incubation was continued for another 48 hrs. Cells were harvested and viral RNA in Huh-7 cells assessed qualitatively by Real-Time PCR.

### RNA Interference

To examine the effects of HCV Core and Cox-2 genes on cellular gene expression, cells were transfected with Core and Cox-2 specific or scrambled siRNAs. Briefly, cells were seeded in 6-well (5×10^5^/well) plates and cultured in CCM until they became 60-80% confluent. Cells were transiently transfected with 10, 20, 40 μM/well of specific siRNAs (Csi27, Csi352 and COXsi either alone or in combination) or scrambled siRNA along with 0.4 μg of HCV Core 3a in serum free media using Lipofectamine™ 2000 (Invitrogen) according to the manufacturer's protocol. After 6 hrs incubation at 37°C in 5% CO_2_, CCM was added to the cells. Cells were then harvested for gene expression and protein analysis after 48 hrs. Protein analysis was carried out for above mentioned experiments in 6-well plates.

For HCV infected experiments, Huh-7 cells were seeded in 6-well plates as described above and cells were infected with HCV infected serum (see Viral Inoculum section for details). To analyze the effect of siRNA on HCV infection, serum-infected Huh-7 cells were seeded after three days of infection in 6 well plates and grown to 80% confluence with 2 ml of standard medium. This was followed by transfection of infected cells with or without 40 μM/well of Csi27 and COXsi siRNA either alone or in combination. Cells were harvested and analyzed for gene expression analysis by Real-Time PCR and viral load determination as described below.

### Cell Proliferation Assay and PGE2 Measurements

Approximately 2×10^3 ^Huh-7 cells were seeded into 96-well tissue culture plates for 24 hrs prior to transfection. Huh-7 cells were transiently transfected with 0.1 μg/well of HCV Core 1a and 3a plasmids with and without Core and Cox-2 siRNA in serum-free media using Lipofectamine™ 2000 (Invitrogen) according to the manufacturer's protocol. After 6 hrs incubation at 37°C in 5% CO_2_, CCM was added to the cells. The MTT colorimetric assay was performed to detect cell proliferation after 24 hrs and 48 hrs of incubation. The absorbance of resulting formazan crystals (solubilized with DMSO) was read at 590 nm on ELISA plate reader. PGE2 levels were also determined in Huh-7 cells using above cell culture/transfection conditions and assayed with the Biotrak Prostaglandin E2 Enzyme Immunoassay system (Amersham Pharmacia Biotech, USA) according to the manufacturer's protocol at 459 nm wavelength.

### Viral Load

Viral RNA was isolated using Gentra RNA isolation kit (Gentra System Pennsylvania, USA) according to the manufacturer's instructions. For viral quantification Sacace HCV quantitative analysis kit (Sacace Biotechnologies Caserta, Italy) was used. Briefly, 10 μl of extracted viral RNA was mixed with an internal control derived from 5'UTR provided by Sacace HCV Real TM Quant kit and subjected to viral quantification using Real-Time PCR Smart Cycler II system (Cepheid Sunnyvale, USA). For protein expression analysis at day 3^rd ^of viral inoculation, cells were washed 3 times before harvesting and Western blotting was performed.

### Real-Time PCR

For gene expression analysis, total RNA from Huh-7 cells transiently transfected with mock, HCV Core 1a and 3a was extracted using TRIzol Reagent (Invitrogen, USA). cDNA was synthesized using 1 μg of total RNA with SuperScript III Kit (Invitrogen, USA). Gene expression analysis was carried out using Real Time PCR ABI 7500 system (Applied Biosystems Inc, USA) with SYBR Green mix (Fermentas, Canada) using gene specific primers (see Table 1). PCR conditions were as follow: 95°C, 30 sec; 58°C, 30 sec and extension at 72°C for 45 sec, 40 cycles. The relative gene expression analysis was carried out using the "SDS 3.1 software" (provided by Applied Biosystems Inc, USA). GAPDH was used as internal control for normalization.

### Western Blotting

Transfected and infected cells were lysed with ProteoJET mammalian cell lysis reagent (Fermentas, Canada). Equal amounts of total proteins were subjected to electrophoresis on 12% SDS-PAGE and electrophoretically transferred to a nitrocellulose membrane using manufacturer's protocol (Bio-Rad, USA). Blots were incubated with specific-monoclonal antibodies of HCV Core, Cox-2, VEGF, p-Akt and GAPDH (Santa Cruz Biotechnology, Inc, USA). Proteins expression was evaluated using Chemiluminescence's detection kit (Sigma Aldrich, USA). For protein quantifications X-ray films were scanned and fold inductions were calculated by densitometer analysis.

### Statistical Analysis

All statistical analysis was performed by using SPSS software (version 16.0, SPSS Inc). Data are presented as mean ± SD. Numerical data were analyzed using student's t-test and ANOVA. *P *value < 0.05 was considered statistically significant.

## List of abbreviations

HCV: Hepatitis C; RNAi: RNA interference; siRNAs: small interfering RNAs; HCC: hepatocellular carcinoma;

## Competing interests

The authors declare that they have no competing interests.

## Authors' contributions

SJ, SK, BI, WA prepared, wrote manuscript and performed lab work. BI, WA, and SH prepared final version of manuscript. SH was also principal investigator and provides support and facilities to complete this work. All authors approved the manuscript.

## Authors' information

Shah Jahan (PhD molecular Biology) and Saba Khaliq (PhD molecular Biology) are both PhD scholars, Bushra Ijaz (M Phil Molecular Biology) and Waqar Ahmad (M Phil Chemistry) are Research Officer, while Sajida Hassan (PhD Molecular Biology) is Principal Investigator at CEMB, University of the Punjab, Lahore

## Supplementary Material

Additional file 1 **Supplemental Results**. This file contains three experimental results as supporting data and has been mentioned in the text. Click here for file

Additional file 2 **Figure S1. Silencing of host cellular gene, Cox-2 show reduction in the expression of genes involved in HCV pathogenesis**. Huh-7 cells were transfected with HCV-3a Core expression vector or mock-treated along with or without 10 μM, 20 μM and 40 μM of Cox-2 siRNAs for 48 hrs. (A) Cox-2 siRNA (COXsi) reduced gene expression of Core induced Cox-2 measured through semi-quantitative PCR. (B) Silencing of Cox-2 gene at protein expression level was determined by Western blot analysis after 48 hrs transfection with mock (M), with and without siRNA (COXsi) and scramble siRNA (SC) in Huh-7 cells. Protein levels for GAPDH gene are also shown as internal control. (C) Effect of silencing of Cox-2 gene on the relative gene expression levels quantified by Real-Time PCR 48 hrs post transfection are shown as fold induction for Cox-2/iNOS/VEGF genes. GAPDH was used as internal control for normalization. Three independent experiments were performed having triplicate samples. Error bars indicate, mean S.D, *p < 0.01 verses Core. Click here for file

Additional file 3 **Figure S2. Combined effect of Cox-2 and HCV 3a Core siRNAs on intracellular PGE2 production and the Huh-7 cells proliferation**. (A) Intracellular level of PGE2 was determined in Core transfected cells with and without Core, Cox-2 and in combination of both siRNAs, using Biotrak prostaglandin Enzyme immunoassay system according to manufacturer's protocol. (B) Inhibition of Huh-7 cells proliferation was observed in Core transfected cells with and without Core, Cox-2 and in combination of both siRNAs by using MTT assay. Three independent experiments were performed having triplicate samples. Error bars indicate mean S.D, *#p < 0.001, *p < 0.01 verses Core. Click here for file

Additional file 4 **Figure S3. HCV 3a sera infection increases expression of genes involved in HCV pathogenesis as compared with HCV 1a sera**. (A) Huh-7 cells were infected with high titer sera samples from HCV patients, either HCV-3a or HCV-1a genotype for 72 hrs. RNA expression levels as relative fold induction to normal sera are shown for Cox-2, iNOS and VEGF genes. (B) The protein expression levels were determined by Western blot analysis from Huh-7 cell lysates infected with HCV-1a (S1a), 3a (S3a) serum compared to normal and effect on Cox-2, VEGF and p-Akt expression using specific antibodies. Protein levels for GAPDH gene are shown as internal control. Three independent experiments with triplicate determinations were performed. Error bars indicate mean S.D, * p < 0.01 verses serum 1a. Click here for file
