# Supplementary material for: Role of HCV Core gene of genotype 1a and 3a and host gene Cox-2 in HCV-induced pathogenesis
Source: Virol J. 2011 Apr 1;8:155. doi: 10.1186/1743-422X-8-155 (PMC3080829; doi:10.1186/1743-422X-8-155)
Supplement: Additional file 1 — Supplemental Results. This file contains three experimental results as supporting data and has been mentioned in the text. [file 1743-422X-8-155-S1.DOC]

**Supplementary Results**

**Result 1S. Effect of silencing of host gene Cox-2 on iNOS, Akt and VEGF genes.**

Cox-2 levels have been shown to correlate with the levels of several key molecules implicated in carcinogenesis such as iNOS, VEGF and Akt, therefore, we determined the effect of Cox-2 silencing on the expression levels of these genes. Transient transfection of *in-vitro* transcribed siRNA (COXsi) in Huh-7 cells after 48 hrs showed reduced RNA expression levels of Cox-2 gene in a dose-dependent manner. Maximum inhibition of 3-fold was observed for Cox-2 at 40µM (Figure 1S.A). Complementary to reduced mRNA levels, Cox-2 protein expression levels also showed 65% reduction when compared with Core-transfected cells and control scramble siRNA (Figure 1S.B). The expression of Cox-2 gene was reduced to the basal levels with its own siRNA, whereas, Cox-2 siRNA treatment decreased the expression levels of iNOS and VEGF genes to 2-fold and 3-fold respectively (Figure 1S.C).

**Result 2S.**

**Combined effect of Cox-2 and HCV-3a Core siRNAs on intracellular PGE2 production and the Huh-7 cells proliferation.**

The ability of Core and Cox-2 specific siRNA were tested to block the accumulation of PGE2 in response to HCV-3a Core gene. Both siRNA (45% and 65% inhibition by Csi27 and COXsi) were capable of blocking the production of PGE2, whereas their combination showed 90% reduction in PGE2 levels in Core-3a transfected Huh-7 cells (Figure 2S.A). The Core induced cell proliferation and PGE2 was also reduced to almost similar levels as mock samples in Huh-7 cells treated with both siRNA in combination (Csi27 and COXsi) (Figure 2S.B).

**Result 3S:**

**Infection of HCV-3a sera and effect on the expression levels of genes involved in HCV-induced pathogenesis**.

We investigated the effect of high titer HCV-1a and 3a sera infection on the expression levels of Cox-2, iNOS, VEGF and Akt genes in Huh-7 cells. HCV-3a sera stimulated the expression of Cox-2 (3-fold), iNOS (2.8-fold) and VEGF (3.4-fold) genes while HCV-1a sera induced Cox-2 (1.6-fold), iNOS (1.2-fold) and VEGF (1.5-fold) genes as compare to normal sera (Figure 3S.A). Similar levels of induction at protein level were also observed for above genes. Lysates from the Huh-7 cells infected with HCV-1a and 3a serum were examined by Western blot analysis using Core, Cox-2, VEGF and p-Akt specific antibodies (Figure 3S.B).
