# Supplementary figures and images for: Role of HCV Core gene of genotype 1a and 3a and host gene Cox-2 in HCV-induced pathogenesis
Source: Virol J. 2011 Apr 1;8:155. doi: 10.1186/1743-422X-8-155 (PMC3080829; doi:10.1186/1743-422X-8-155)

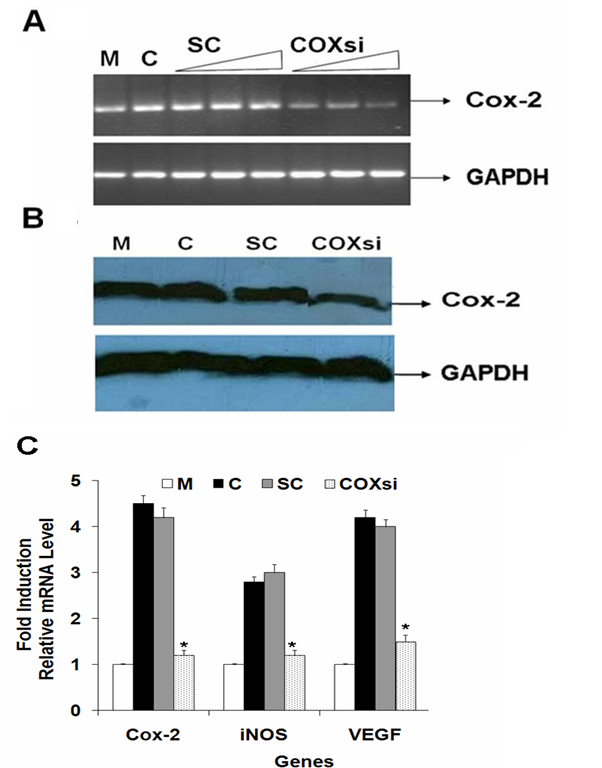

Supplement: Additional file 2 — Figure S1. Silencing of host cellular gene, Cox-2 show reduction in the expression of genes involved in HCV pathogenesis. Huh-7 cells were transfected with HCV-3a Core expression vector or mock-treated along with or without 10 μM, 20 μM and 40 μM of Cox-2 siRNAs for 48 hrs. (A) Cox-2 siRNA (COXsi) reduced gene expression of Core induced Cox-2 measured through semi-quantitative PCR. (B) Silencing of Cox-2 gene at protein expression level was determined by Western blot analysis after 48 hrs transfection with mock (M), with and without siRNA (COXsi) and scramble siRNA (SC) in Huh-7 cells. Protein levels for GAPDH gene are also shown as internal control. (C) Effect of silencing of Cox-2 gene on the relative gene expression levels quantified by Real-Time PCR 48 hrs post transfection are shown as fold induction for Cox-2/iNOS/VEGF genes. GAPDH was used as internal control for normalization. Three independent experiments were performed having triplicate samples. Error bars indicate, mean S.D, *p < 0.01 verses Core. [file 1743-422X-8-155-S2.BMP]

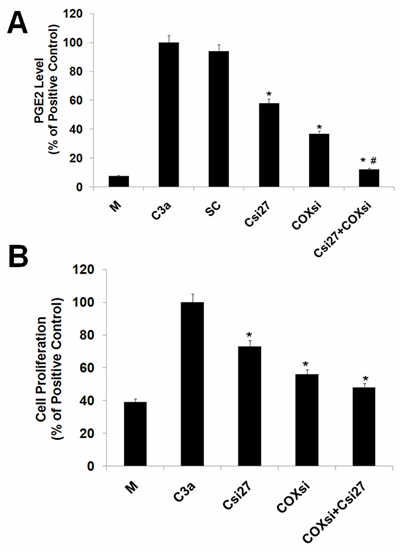

Supplement: Additional file 3 — Figure S2. Combined effect of Cox-2 and HCV 3a Core siRNAs on intracellular PGE2 production and the Huh-7 cells proliferation. (A) Intracellular level of PGE2 was determined in Core transfected cells with and without Core, Cox-2 and in combination of both siRNAs, using Biotrak prostaglandin Enzyme immunoassay system according to manufacturer's protocol. (B) Inhibition of Huh-7 cells proliferation was observed in Core transfected cells with and without Core, Cox-2 and in combination of both siRNAs by using MTT assay. Three independent experiments were performed having triplicate samples. Error bars indicate mean S.D, *#p < 0.001, *p < 0.01 verses Core. [file 1743-422X-8-155-S3.BMP]

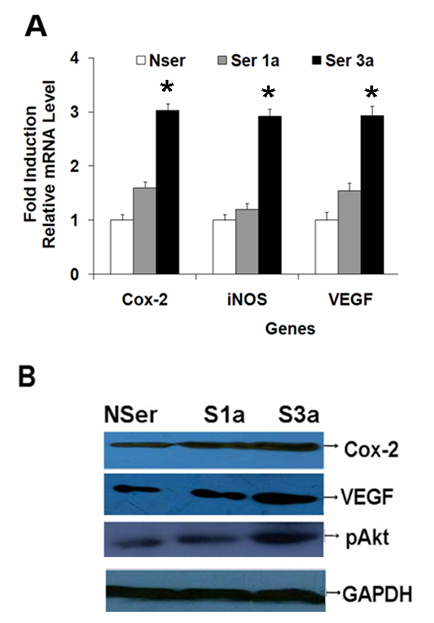

Supplement: Additional file 4 — Figure S3. HCV 3a sera infection increases expression of genes involved in HCV pathogenesis as compared with HCV 1a sera. (A) Huh-7 cells were infected with high titer sera samples from HCV patients, either HCV-3a or HCV-1a genotype for 72 hrs. RNA expression levels as relative fold induction to normal sera are shown for Cox-2, iNOS and VEGF genes. (B) The protein expression levels were determined by Western blot analysis from Huh-7 cell lysates infected with HCV-1a (S1a), 3a (S3a) serum compared to normal and effect on Cox-2, VEGF and p-Akt expression using specific antibodies. Protein levels for GAPDH gene are shown as internal control. Three independent experiments with triplicate determinations were performed. Error bars indicate mean S.D, * p < 0.01 verses serum 1a. [file 1743-422X-8-155-S4.BMP]
